# Supplementary material for: The genome sequence of the starved wood-sedge, Carex depauperata Curtis ex Woodw. (Poales: Cyperaceae)
Source: Wellcome Open Res. 2026 Mar 18;11:165. [Version 1] doi: 10.12688/wellcomeopenres.26113.1 (PMC13069380; doi:10.12688/wellcomeopenres.26113.1)
Supplement: Peer review history

# Reviewer report: Reviewer response for version 1

**DOI:** 10.21956/wellcomeopenres.28756.r151039

**Published:** 2026-04-15

**Authors:** Harpreet Kaur

**Affiliations:** 1 University of Minnesota, St. Paul, Minnesota, USA

**General Assessment**

 The manuscript titled *"The genome sequence of the starved wood-sedge, Carex depauperata* Curtis ex Woodw. (Poales: Cyperaceae)" presents a genome assembly based on PacBio HiFi and Hi-C sequencing data and is clearly written overall. The following minor issues should be addressed prior to full acceptance:

**Specific Comments**

**1. Repeat Content Estimation** The repeat content percentage reported is derived from k-mer frequency analysis using GenomeScope2, which operates on raw, unassembled reads. While this provides a useful pre-assembly genomic survey, it is not an accurate reflection of repeat content in the final assembled genome. The authors should either clearly qualify this value as a pre-assembly estimate, or preferably, report repeat content derived from annotation of the assembled genome using tools such as RepeatMasker, EDTA, or equivalent pipelines.

**2. BUSCO Score Inconsistency** The BUSCO completeness values cited in the main text do not appear to be consistent with those reported in Table 4. The authors should carefully verify and reconcile these values to ensure internal consistency throughout the manuscript.

**3. Organellar Genome Sizes** The sizes of the mitochondrial and plastid genomes are currently mentioned only in the abstract. Given their relevance to the overall genomic characterization, these values should also be reported in the appropriate section of the main text.

Are sufficient details of methods and materials provided to allow replication by others?

Yes

Is the rationale for creating the dataset(s) clearly described?

Yes

Are the datasets clearly presented in a useable and accessible format?

Yes

Are the protocols appropriate and is the work technically sound?

Yes

Reviewer Expertise:

Comparative Genomics

I confirm that I have read this submission and believe that I have an appropriate level of expertise to confirm that it is of an acceptable scientific standard, however I have significant reservations, as outlined above.

# Reviewer report: Reviewer response for version 1

**DOI:** 10.21956/wellcomeopenres.28756.r151675

**Published:** 2026-04-10

**Authors:** Jian-Qiang Zhang

**Affiliations:** 1 Shaanxi Normal University, Xi'an, China

The authors sequenced and assembled the genome of *Carex depauperata* (starved wood-sedge; Cyperaceae) using a combination of HiFi and Hi-C data. The final assembly has a total length of 702.60 Mb, with 99.61% of sequences anchored to 22 chromosomal pseudomolecules, indicating a generally high-quality assembly. This genomic resource will be valuable for future studies in *Carex* and Cyperaceae. I offer the following suggestions to further strengthen the manuscript:

 1. In Figure 3, the prominent grey line between Chr 17 and Chr 18 is quite noticeable and somewhat unexpected. It would be helpful if the authors could clarify whether this represents a biological signal or a visualization artifact, and adjust the figure if necessary to improve clarity.

 2. The BUSCO results show a relatively high proportion of missing genes (22.1%), which may indicate limited completeness. While the use of the poales_odb10 dataset is appropriate, it may be somewhat stringent. The authors might consider also evaluating completeness using the embryophyta_odb10 dataset to provide a complementary perspective.

 3. Including an additional metric such as the LTR Assembly Index (LAI) would further strengthen the assessment of genome quality and provide a more comprehensive evaluation of assembly integrity.

 4. Given that many *Carex* species possess holocentric chromosomes, it would be interesting if the authors could comment on whether *C. depauperata* shares this feature, or discuss relevant evidence if available.

Are sufficient details of methods and materials provided to allow replication by others?

Yes

Is the rationale for creating the dataset(s) clearly described?

Yes

Are the datasets clearly presented in a useable and accessible format?

Yes

Are the protocols appropriate and is the work technically sound?

Yes

Reviewer Expertise:

Plant systematics and evolution

I confirm that I have read this submission and believe that I have an appropriate level of expertise to confirm that it is of an acceptable scientific standard, however I have significant reservations, as outlined above.

# Reviewer report: Reviewer response for version 1

**DOI:** 10.21956/wellcomeopenres.28756.r151033

**Published:** 2026-04-08

**Authors:** Roberto Biello

**Affiliations:** 1 University of Copenhagen Globe Institute (Ringgold ID: 552766), Copenhagen, Capital Region of Denmark, Denmark

The authors present a high-quality, chromosome-level genome assembly for the starved wood-sedge, *Carex depauperata*, using PacBio HiFi and Hi-C sequencing. This represents a valuable genomic resource for a species on the brink of extinction in Britain and Ireland. The assembly achieves a high QV score and 99.61% of the sequences were assigned to 22 chromosomal-level scaffolds. The methods are clearly described, and all associated data are publicly available. Overall, this is a well-executed genome note.

 Comments:

 - The manuscript reports a flow cytometry-based estimate of ~860 Mb, yet this appears to diverge from the k-mer analysis and the final assembly length. The authors should explicitly discuss this discrepancy. Furthermore, please compare these findings with previous estimates for related species, specifically Lipnerová et al. (Refer to reference no. 1), to provide broader context for *Carex* genome evolution.

 - The reported single-copy BUSCO score is unexpectedly low. The authors should provide possible biological or technical explanations.

Are sufficient details of methods and materials provided to allow replication by others?

Yes

Is the rationale for creating the dataset(s) clearly described?

Yes

Are the datasets clearly presented in a useable and accessible format?

Yes

Are the protocols appropriate and is the work technically sound?

Yes

Reviewer Expertise:

Evolutionary biology, Genome assembly and annotation

I confirm that I have read this submission and believe that I have an appropriate level of expertise to confirm that it is of an acceptable scientific standard.

## References

[1] : Evolution of genome size in Carex (Cyperaceae) in relation to chromosome number and genomic base composition. *Annals of Botany* .2013;111(1) : 10.1093/aob/mcs239 79-94 10.1093/aob/mcs239 PMC352365223175591

# Reviewer report: Reviewer response for version 1

**DOI:** 10.21956/wellcomeopenres.28756.r151034

**Published:** 2026-04-07

**Authors:** Abdoallah Sharaf

**Affiliations:** 1 University of Konstanz, Konstanz, Germany

The manuscript presents a chromosome-level genome assembly for the Endangered plant *Carex depauperata* as part of the Darwin Tree of Life (DToL) project. The final assembly spans 702.60 Mb, with 99.61% of the sequence scaffolded into 22 chromosomal pseudomolecules (scaffold N50 = 32.58 Mb). Additional assemblies include eight mitochondrial sequences and a plastid genome of 215.32 kb. The methods are well documented and largely follow DToL/Welcome Sanger Institute best practices, with transparent data deposition in the INSDC.

 Overall, the assembly shows strong contiguity and high consensus quality (QV ≈ 70 for the primary haplotype). However, several aspects of quality assessment, result presentation, and justification require clarification or supplementation to fully support its status as a high-quality reference genome.

**Major points (requiring author response):**

1. **BUSCO completeness reporting needs clarification and context** In the manuscript, BUSCO completeness is reported as 75.6% complete using the poales_odb10 lineage (single-copy 68.2%, duplicated 7.3%). However, the BlobToolKit viewer shows additional runs: 87.9% for lilopsida_odb10 and **95.6% for embryophyta_odb10**. The poales_odb10 score is noticeably low compared to EBP expectations (>90% complete genes, preferably >90% single-copy with <5% duplicated). The embryophyta score is much stronger and more appropriate for broad plant genome assessment. **Recommendation:** Clearly present all three BUSCO results in the main text and Table 4 (or a revised table), specify which lineage is considered primary, and explain the substantial difference across lineages. A brief discussion of why the poales-specific score is lower (e.g., known limitations of the current poales_odb10 database for Cyperaceae or lineage-specific gene content) would be helpful.
2. **Alignment with Earth BioGenome Project (EBP) assembly standards** The manuscript claims an EBP summary metric of **6.C.Q70**. While contig N50 (3.88 Mb), scaffold N50, chromosomal assignment (>99%), k-mer completeness (>98%), and QV (~70) perform well, the low poales_odb10 BUSCO score means the assembly does not fully meet the latest EBP reference standards (Version 7.0, January 2026), which recommend >90% single-copy conserved genes. **Recommendation:** Revise Table 4 to transparently indicate which EBP criteria are fully met and provide justification for any shortfalls, including reference to the more appropriate embryophyta BUSCO result.
3. **Flow cytometry genome size estimate is inadequately presented** the 1C-value of 0.88 pg (≈860 Mb) is stated but lacks any supporting histogram, peak CV values, or detailed discussion. This estimate is ~20% higher than both the GenomeScope2 prediction (723.71 Mb) and the assembled size (702.60 Mb). **Recommendation:** Include the flow cytometry histogram as a supplementary figure (clearly labelled with peak positions and CVs) and briefly discuss possible biological or technical reasons for the discrepancy.
4. **DNA barcoding results are not reported** The text states that species identification was verified via DNA barcoding following the DToL framework (Twyford et al., 2024), with comparison to BOLD and GenBank. However, no actual results are provided (e.g., BOLD Process IDs, GenBank accessions for rbcL/matK/ITS, similarity percentages, or confirmation of identity). **Recommendation:** Report the barcoding outcomes explicitly in the relevant section or as a supplementary table. This is a standard expectation for DToL genome notes to confirm taxonomic accuracy.
5. **Hi-C contact map quality** The Hi-C contact map (Figure 3) shows noticeable off-diagonal background signal, which is not discussed. **Recommendation:** Address the background noise level, explain the effect of the manual curation steps (five breaks and seven joins), and comment on the resulting confidence in the chromosomal pseudomolecules.

**Minor points:**

- Reconcile minor inconsistencies in reported chromosomal assignment percentages (99.61% vs. 99.77%).
- Briefly contextualize GenomeScope2 estimates (0.16% heterozygosity, 43.42% repeats) relative to assembly performance.
- Strengthen the introduction/discussion by noting specific potential uses of this genome (e.g., for conservation of the tiny British/Irish populations or evolutionary studies in the depauperate *Carex* lineage).

**Overall assessment:**

 This is a technically solid DToL genome note with excellent scaffolding, high QV, and good transparency. The main weaknesses lie in incomplete or unclear reporting of key quality metrics (BUSCO across lineages, flow cytometry, DNA barcoding) and limited justification against EBP standards. Once the authors address these points — particularly by providing the missing barcoding results, better presenting the flow cytometry data, and clearly contextualizing the BUSCO scores — the manuscript will be significantly strengthened and suitable for indexing.

Are sufficient details of methods and materials provided to allow replication by others?

Partly

Is the rationale for creating the dataset(s) clearly described?

Yes

Are the datasets clearly presented in a useable and accessible format?

Yes

Are the protocols appropriate and is the work technically sound?

Partly

Reviewer Expertise:

Genomics, bioinformatics, plant genome assembly, and biodiversity research.

I confirm that I have read this submission and believe that I have an appropriate level of expertise to confirm that it is of an acceptable scientific standard, however I have significant reservations, as outlined above.
